# Endoplasmic Reticulum Stress Response in Arabidopsis Roots

**DOI:** 10.3389/fpls.2017.00144

**Published:** 2017-03-01

**Authors:** Yueh Cho, Kazue Kanehara

**Affiliations:** ^1^Institute of Plant and Microbial Biology, Academia SinicaTaipei, Taiwan; ^2^Molecular and Biological Agricultural Sciences Program, Taiwan International Graduate Program, Academia Sinica and National Chung-Hsing UniversityTaipei, Taiwan; ^3^Graduate Institute of Biotechnology, National Chung-Hsing UniversityTaichung, Taiwan; ^4^Biotechnology Center, National Chung-Hsing UniversityTaichung, Taiwan; ^5^Muroran Institute of TechnologyMuroran, Japan

**Keywords:** *Arabidopsis thaliana*, ER stress response, root, stress response, UPR

## Abstract

Roots are the frontier of plant body to perceive underground environmental change. Endoplasmic reticulum (ER) stress response represents circumvention of cellular stress caused by various environmental changes; however, a limited number of studies are available on the ER stress responses in roots. Here, we report the tunicamycin (TM) -induced ER stress response in Arabidopsis roots by monitoring expression patterns of immunoglobulin-binding protein 3 (BiP3), a representative marker for the response. Roots promptly responded to the TM-induced ER stress through the induction of similar sets of ER stress-responsive genes. However, not all cells responded uniformly to the TM-induced ER stress in roots, as BiP3 was highly expressed in root tips, an outer layer in elongation zone, and an inner layer in mature zone of roots. We suggest that ER stress response in roots has tissue specificity.

## Introduction

Roots are the frontier of plant body to perceive underground environmental change. In response to environmental stimuli, a crucial set of molecular processes is induced that maintains cellular homeostasis and thus circumvents fatal defects caused by the stresses. Among various organelles involved in the cellular homeostasis, the ER plays a decisive role in protein folding and secretion.

The ER is the gateway for the eukaryotic protein secretory pathway. Secretory proteins are translocated into the ER and enter protein-folding cycles to fold and assemble themselves (Anelli and Sitia, 2008). Only properly folded proteins are allowed to leave the ER by a surveillance system, collectively termed the ER quality control. The ER quality control is well-conserved molecular mechanisms among eukaryotic cells including animals, yeasts and plants (Iwata and Koizumi, 2005, 2012; Howell, 2013). When aberrant proteins are accumulated in the ER, the ER quality control recognizes these aberrant proteins and responds to maintain the ER homeostasis using multiple strategies such as UPR and ER-associated degradation (ERAD) (Kanehara et al., 2007; Walter and Ron, 2011; Ruggiano et al., 2014). The ER membrane-localized ribonuclease inositol-requiring enzyme 1 (IRE1) is one of major signal transducers in the UPR. The IRE1 senses protein-folding status in the ER and transmits signals into the nuclei by catalyzing unconventional cytoplasmic splicing of *bZIP60* mRNA in plants (*XBP1* in mammals and *HAC1* in yeasts) followed by activation of UPR target genes including a molecular chaperone BiP (Nagashima et al., 2011; Walter and Ron, 2011). BiP is one of the most abundant chaperones in the ER lumen and is thought to bind nascent peptides to prevent protein aggregation. BiPs belong to the heat shock protein 70 family that binds ATP and operates in conjunction with J-domain-containing proteins (J proteins) (Fewell et al., 2001). Arabidopsis genome encodes three *BiP* genes, *BiP1* (At5g28540), *BiP2* (At5g42020), and *BiP3* (At1g09080) (Noh et al., 2003). *BiP1* and *BiP2* encode ubiquitously expressed proteins whose amino acid sequences are 99% identical to each other. *BiP3* encodes a less conserved protein, whose expression is limited under ER stress conditions in young seedlings (Noh et al., 2003). BiPs are also master regulators of the ER stress response in Arabidopsis (Srivastava et al., 2013). Recent studies showed that *BiP3* functions in pollen development and female gametogenesis (Maruyama et al., 2014, 2015).

Extensive studies across different model organisms have revealed details of molecular mechanisms underlying the ER stress response. In plant research, however, ER stress response is an emerging subject despite its high relevance to general plant stress response studies (Liu and Howell, 2010). In addition to the conserved molecular mechanisms, recent studies have suggested that plants may have plant-specific ER stress responses, involving heterotrimeric G proteins and phosphoinositide signaling (Wang et al., 2007; Chen and Brandizzi, 2012; Kanehara et al., 2015b). Our understanding about the molecular mechanisms of the ER stress response in plants has been based mostly on the studies using whole Arabidopsis seedlings. Because root biomass is marginal to the total seedling biomass, effect of root-specific changes may be diluted to be non-measureable in the whole seedling sample even if the root and shoot differentially responds to the ER stress. In fact, previous studies explored ER stress responses in roots of various plant species. For example, the tissue-specific transcriptional regulation of soybean *BiPs, gsBiP6* and *gsBiP9* in transgenic *Nicotiana tabacum* plants was reported using *gsBiPs* promoter-GUS chimeric reporter genes (Buzeli et al., 2002). In roots of *Pisum sativum*, the expression levels of *BiP-D*, *bZIP28*, and *bZIP60* were elevated during tungsten treatment, which is known to affect plant growth (Adamakis et al., 2011). In addition, rice *OsBiPs* and its co-chaperones, *OsERdjs*, were transcriptionally upregulated under the ER stress conditions in roots of rice seedlings (Ohta et al., 2013). However, little is known about a detailed molecular mechanism of ER stress response in roots despite that root is an important organ to perceive environmental stresses. Although tissue specificity of the UPR in plants has been investigated in the gametophyte development (Maruyama et al., 2010, 2014; Deng et al., 2013), it remains elusive whether an individual cells of multicellular organisms responds uniformly or differentially to the ER stress caused by external environmental stresses. The Arabidopsis root is an excellent model to investigate tissue type- and cell type-specific response *in vivo* because it is a transparent organ and each tissue/cell type has been characterized well.

In an effort to explore the ER stress response in the plant root system and address tissue-specific response in an intact multicellular organism, current study investigated ER stress response in Arabidopsis roots. To monitor the ER stress responses, a well-described UPR gene *BiP3* has been employed because of the extremely low expression under non-stress condition but acute induction upon ER stress (Noh et al., 2003; Srivastava et al., 2013; Cho et al., 2015). Based on the time-course observation of stable transgenic plant expressing the *ProBiP3:BiP3-GUS-HDEL* or *ProBiP3:mRFP*, we found that BiP3 was differentially expressed in root under the ER stress condition: the root tip including columella, outer layers in elongation zone and inner layers in mature zone were highly responsive to the ER stress. Our results suggest that the ER stress response has tissue-type and cell-type specificity and not all cells may respond uniformly to the ER stress in the Arabidopsis roots.

## Materials and Methods

### Plant Materials and Growth Condition

Arabidopsis plants (Columbia-0 ecotype) were grown under continuous light at 22°C. Murashige and Skoog (MS) media was used at half-strength concentration for plant culture (Murashige and Skoog, 1962). Seeds of *bip3-1* (SALK_024133) were obtained from Nottingham Arabidopsis Stock Centre (NASC). Homozygous T-DNA mutant plants were isolated by PCR-based genotyping with the specific primers (KK200/KK201, LB1.3/KK201). Position of T-DNA insertion was determined by sequencing to be located within the protein coding sequence of *BiP3* (**Figure 3A**). For TM treatment, seedlings were immersed in liquid MS media containing 5 μg/ml TM for indicated time. For detection of aggregated proteins, seedlings were immersed in liquid MS media containing 10 mM MG-132 for 16 h. DMSO was used as negative controls for both TM and MG-132 treatments.

### Sequence Alignment of BiPs

The amino acid sequences of three BiP isoforms were adopted from TAIR database (protein accession numbers: BiP1: 1009129411, BiP2: 1009134007, and BiP3: 5019479994). A multiple alignment of the protein sequences for BiPs was assembled using ClustalW^1^ and BoxShade^2^.

### Plasmid Vector Construction and Plant Transformation

A 4 kbp fragment of the genomic sequence for *BiP3* was amplified by PCR with oligonucleotide primers KK131 and KK132, and cloned into the pENTR/D-TOPO plasmid vector (Invitrogen, Carlsbad, CA, USA) to obtain pCC38. To create the GUS reporter construct (*ProBiP3:BiP3-GUS-HDEL*), *Sma*I site was inserted at the position immediately before the ER retention sequence HDEL of BiP3 by PCR-based site directed mutagenesis with primer KK152 (Sawano and Miyawaki, 2000). Then, a GUS cassette was inserted into the *Sma*I site to produce pCC71, which was recombined to a pBGW destination vector by use of LR Clonase (Karimi et al., 2005). The resulting pCC67 was transformed into wild-type (WT) plants via Agrobacterium GV3101-mediated gene transformation. Twenty-four transformed plants were selected by spraying 0.1% Basta solution to the seedlings on soil. The T1 seeds were screened by Basta, and the resistant plants harboring *ProBiP3:BiP3-GUS-HDEL* were selected by PCR-based genotyping with primers (KK98/KK200). *ProBiP3:BiP3-GUS-HDEL* line No. 17 was selected as a representative line for observation. For the fluorescent reporter construct (*ProBiP3:mRFP*), the 0.9 kbp promoter region of *BiP3* was amplified with primers KK131 and KK172, and cloned into pENTR/D-TOPO plasmid vector (Invitrogen, Carlsbad, CA, USA) to obtain pCC76. This was recombined into a destination vector pGWB653 (Nakamura et al., 2010) by use of LR Clonase and the resulting plasmid pCC79 was transformed into WT plants via Agrobacterium GV3101-mediated gene transformation. Then, 16 plants were selected by spraying 0.1% Basta solution to the seedling on soil. The T2 seeds were screened by Basta, and the resistant plants harboring *ProBiP3:mRFP* were selected by PCR-based genotyping with primers (KK202/KK133). *ProBiP3: mRFP* line No. 11 was selected as a representative line and used for observation by confocal laser-scanning microscopy. The sequence of primers used were listed in Supplementary Table S1.

### Quantitative RT-PCR (qRT-PCR)

Quantitative RT-PCR analysis was performed as previously described using total RNA was isolated from 7-day-old seedlings (Lin et al., 2015). The means and standard deviations of ΔΔCT were calculated from three independent biological replicates for whole seedlings. Six independent biological replicates were used for roots. The primers used for qRT-PCR are listed in Supplementary Table S1.

### Histochemical GUS Staining

Gus staining was performed as previously described by Kanehara et al. (2015a). Briefly, seedling samples were immersed in GUS staining solution (10 mM EDTA, 5 mM potassium ferricyanide, 5 mM potassium ferrocyanide, 0.1% [w/v] Triton X-100, and 0.5 mg/ml X-Gluc [5-bromo-4-chloro-3-indolyl-β-D-glucuronide] in 100 mM phosphate buffer), and incubated at 37°C. Then, the reaction was stopped by replacing the solution with 70% ethanol. For colored tissues, pigments were removed by immersing the tissue in 6:1 (v:v) ethanol : acetic acid. The images were obtained using a stereomicroscope (Zeiss Stemi 2000) equipped with a Nikon D7000 camera and an upright microscope (Zeiss Axio Imager A2) equipped with a Canon EOS 500D camera.

### Preparation of Anti-BiP3 Antibody and Immunoblotting

To avoid a cross-reaction with BiP1 and BiP2, which show high amino acid similarity to BiP3, a polypeptide consisting of the C-terminal 19 amino acid residues of BiP3, VYEKTEGENEDDDGDDHDE, was synthesized and used to raise an anti-BiP3 polyclonal antibody in rabbits (LTK BioLaboratories, Taoyuan, Taiwan). For immunoblotting, total cell lysate from seedlings or roots was extracted in a lysis buffer [50 mM Tris-HCl (pH6.8), 2% SDS, 10 mM β-mercaptoethanol, 1% v/v protease inhibitor cocktail (Sigma)]. Protein samples were separated by 10% acrylamide SDS-PAGE and transferred to a polyvinylidene difluoride membrane for immunoblotting with rabbit polyclonal anti-BiP3 antibodies; 1:2,000, followed by goat anti-rabbit IgG peroxidase conjugates (Santa Cruz); 1:10,000. BiP3 proteins were visualized by use of chemiluminescence detection reagent (SuperSignal West Pico, Pierce) and Image Quant LAS4000 (GE Healthcare). SDS-PAGE gel was also stained with 0.1% Coomassie Brilliant Blue for 1 h at room temperature.

### Confocal Laser-Scanning Microscopy

Fluorescence of mRFP in seedlings of *ProBiP3:mRFP* was observed under a microscope (LSM 510 Meta; Carl Zeiss) equipped with Objectives C-Apochromat 40×/1.2-numerical aperture (NA) and C-Apochromat 63×/1.2-NA. Images were captured using an LSM 510 v3.2 confocal microscope (Carl Zeiss) with filter (543-nm laser, band-pass 560–615 nm). Cell boundaries were visualized by differential interference contrast (DIC) images.

### Detection of Aggregated Protein

Aggregated proteins were stained by Proteostat^®^ Aggresome Detection Kit (Enzo: ENZ-51035) according to manufacturer’s instruction with a slight modification. Briefly, seedlings after the chemical treatments were fixed in 4% paraformaldehyde in the assay buffer (Proteostat^®^ Aggresome Detection Kit, Enzo) for 30 min at room temperature, which was then washed by phosphate-buffered saline (PBS) three times. Seedlings were transferred into a permeabilizing solution (0.5% Triton X-100, 3 mM EDTA, pH 8) for 30 min. After washing with PBS buffer, seedlings were incubated with Proteostat^®^ dye (Proteostat^®^ Aggresome Detection Kit, Enzo) at 1:5000 dilution for 1 h in the dark.

### Immunolocalization Analysis of BiP3 and Aggregated Proteins

After chemical treatments, seedlings were fixed in 4% paraformaldehyde (Merck) in PBS for 90 min with vacuum dry. To break down cell walls, the seedlings were incubated with 1.5% Driselase (Sigma) in PBS at 37°C for 50 min. For plasma membrane penetration, the samples were incubated with a PBS solution containing 3% IGEPAL CA-630 (Sigma) and 10% DMSO for 30 min at room temperature. To decrease non-specific binding, samples were blocked in PBS containing 3% BSA for 3 h at room temperature. After overnight incubation with anti-BiP3 antibodies (1:100) at 4°C, samples were washed with PBS six times, and incubated with secondary antibody, goat anti-rabbit Alexa Fluor 488 (1:1,000, Life Technologies) for 3 h at room temperature. To detect aggregated proteins, samples were incubated with Proteostat^®^ dye (Proteostat^®^ Aggresome Detection Kit, Enzo; 1:5000) for 1 h at room temperature. After three times washing by PBS, samples were mounted in a drop of 9:1 (v/v) glycerol: PBS, and observed under a microscope (LSM 510 Meta; Carl Zeiss) equipped with objectives C-Apochromat 40×/1.2-NA and C-Apochromat 63×/1.2-NA. Images were captured using an LSM 510 v3.2 confocal microscope (Carl Zeiss) with filter (488-nm laser, band-pass 505–530 nm) for BiP3, and with filter (543-nm laser, band-pass 560–615 nm) for aggregated proteins. Cell boundaries were visualized by DIC images.

## Results

### Observation of the ER Stress Response in Roots via the BiP3-GUS Reporter System

**FIGURE 1:**
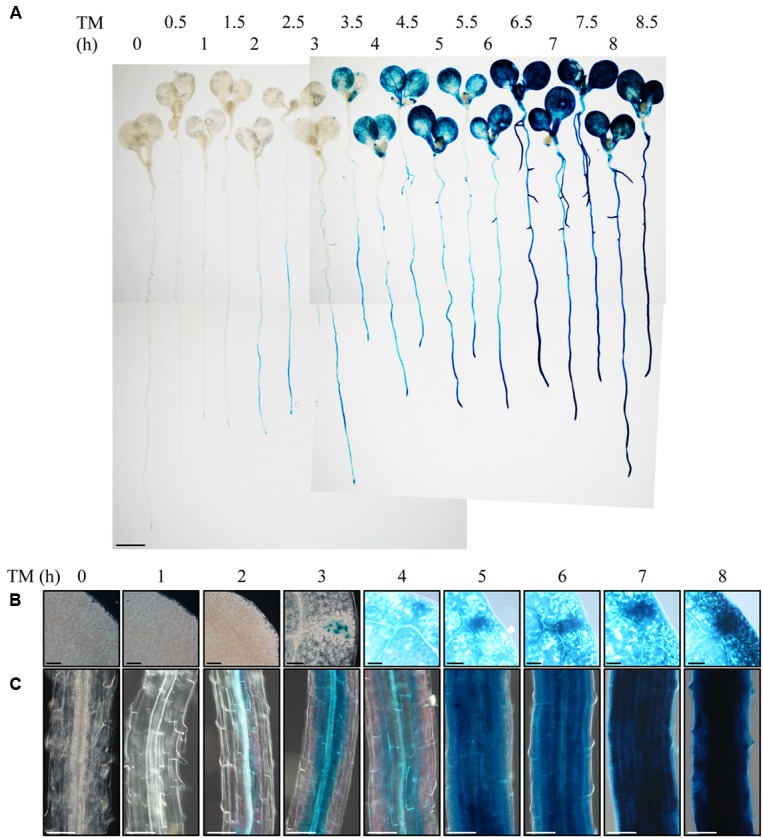
**Observation of the ER stress response in root with the *BiP3-GUS* reporter system.** Seven-day-old seedlings of *ProBiP3:BiP3-GUS* were treated with 5 μg/ml TM for time indicated and GUS staining was performed. **(A)** Whole seedlings or magnified view of the cotyledon **(B)**, and the maturation zone of roots **(C)**. Scale bars: 1 mm **(A)**; 100 μm **(B,C)**.

To observe the ER stress response in roots of intact plants, we employed Arabidopsis *BiP3* gene as a reporter. *BiP3* is a widely used marker gene for the ER stress response, whose expression is extremely low at either RNA or protein levels under non-stress conditions but is highly up-regulated upon the ER stress in Arabidopsis young seedlings (Noh et al., 2003; Cho et al., 2015). We established a transgenic Arabidopsis plant that stably expresses BiP3-GUS fusion protein containing the ER retention signal (HDEL) for ER localization, which is driven by the own promoter (*ProBiP3:BiP3-GUS-HDEL* in WT background). We treated 7-day-old seedlings of the *ProBiP3:BiP3-GUS-HDEL* plants with TM for 0 to 8.5 h and observed the expression of GUS reporter by histochemical staining (**Figure 1A**). TM inhibits protein *N*-glycosylation and thus induces ER stress (Helenius and Aebi, 2004). As can be seen, GUS staining emerged after 2-h treatment mainly at vascular bundles in roots and root tip but no staining in leaves (**Figures 1A,C**). At 3 h, an obvious staining appeared first in hydathodes of leaves (**Figures 1A,B**), which was extended to the entire cotyledons at 4 h (**Figures 1A,B**). In roots, the GUS expression was enhanced from the vasculature to the outer layer by extending the duration of TM treatment (**Figure 1C**). Despite the exogenous chemical treatment by TM, the first GUS expression was detected in the root vasculatures, an innermost tissue. This observation suggests that roots respond to TM treatment more rapidly than leaves, and that the vasculatures and root tips are the initial sites of TM-induced ER stress response in Arabidopsis roots.

### Expression Patterns of UPR Genes in Roots

**FIGURE 2:**
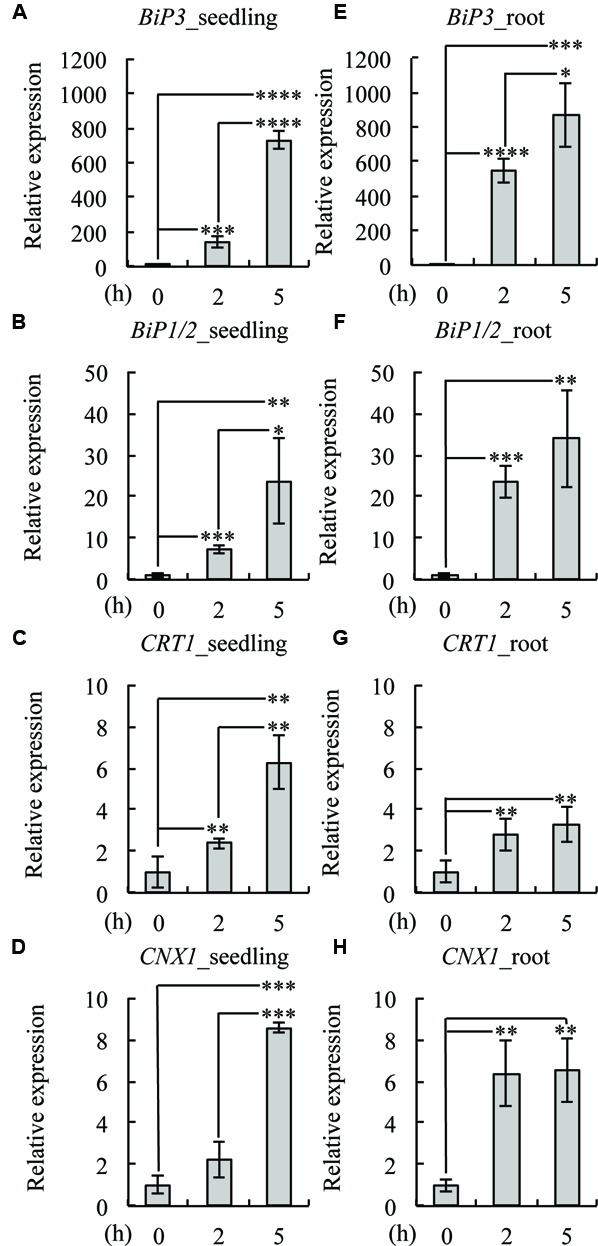
**Expression patterns of UPR genes in roots.** Quantitative RT-PCR analysis of UPR-responsive genes was performed using 7-day-old seedlings of the wild-type (WT) plant treated with dimethyl sulfoxide (DMSO) as mock (0 h) or 5 μg/ml TM for 2 or 5 h. Whole seedlings or roots were collected for RNA extraction and cDNA synthesis. Transcript levels of *BiP3* **(A,E)**, *BiP1/2* **(B,F)**, calreticulin (*CRT1*; **C,G**), and calnexin (*CNX1*; **D,H**) were quantified. The expression of the sample at 0 h was set to 1. Data were averaged by three technical replicates in the same run and three biological replicates in separate runs with SD. Asterisks indicate significance by Student *t-*test (^∗^, *P* < 0.05; ^∗∗^, *P* < 0.01; ^∗∗∗^, *P* < 0.001; ^∗∗∗∗^, *P* < 0.0001).

To investigate whether the same set of UPR genes is induced by TM treatment in roots, we treated 7-day-old WT seedlings with TM and compared the gene expression of *BiP3*, *BiP1/2*, *CALRETICULIN* (*CRT1*: At1g56340), and *CALNEXIN* (*CNX1*: At5g61790) at 2 and 5 h after the TM treatment by qRT-PCR (**Figure 2**). The expression of all of these genes was induced both in the whole seedlings (**Figures 2A–D**) and roots (**Figures 2E–H**) upon TM treatment. These data suggest that a similar set of UPR genes was induced by TM treatment in roots as compared to the whole seedlings. Notably, however, some minor difference was observed including higher fold increase in *BiP3, BiP1/2* and *CNX1* at 2 h (**Figures 2A,B,D,E,F,H**). These results are consistent with the GUS reporter assay (**Figure 1**), in which roots robustly responded upon TM treatment.

### Accumulation of BiP3 Protein by TM Treatment

**FIGURE 3:**
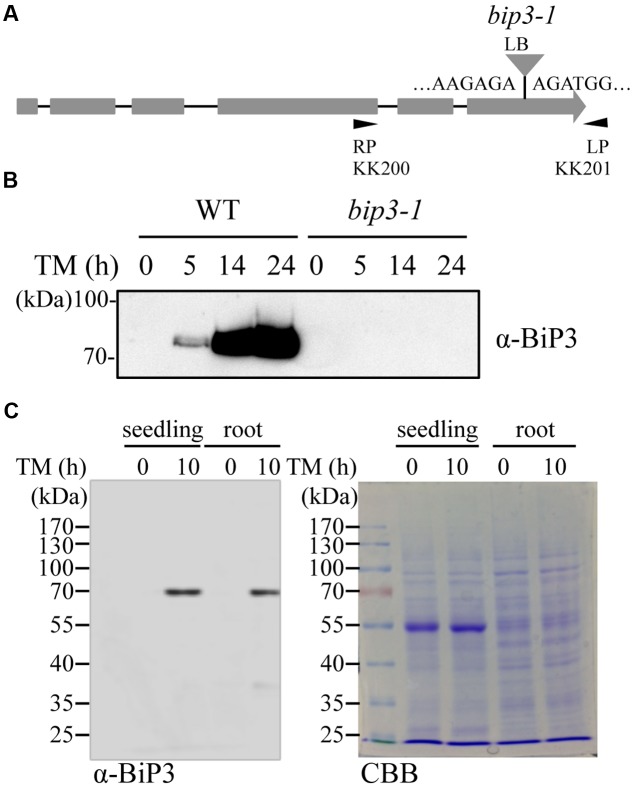
**Accumulation of BiP3 protein by TM treatment in roots. (A)** Schematic representation of the gene structure of *BiP3* and T-DNA position of *bip3-1* mutant. Gray boxes represent exons, and the positions of T-DNA insertion was shown as triangle. **(B)** Immunoblot analysis of BiP3 protein in WT and *bip3-1* seedlings in response to TM. Total protein was extracted from 7-day-old WT or *bip3-1* seedlings treated with 5 μg/ml TM for the indicated time. **(C)** Immunoblot analysis of BiP3 protein in roots of WT seedlings in response to TM. WT seedlings were treated as described in **(B)**, and samples were collected. Total proteins were subjected to immunoblot analysis for BiP3 protein (left panel), and CBB staining (right panel).

To detect endogenous BiP3 protein in WT plants, we produced polyclonal anti-BiP3 antibodies against a synthetic peptide corresponding to 19 amino acid residues at C-terminus of BiP3 protein because of low sequence similarity of this region with those in BiP1 and BiP2 (**Supplementary Figure S1**). To examine the specificity of the antibodies, we extracted total protein from 7-day-old seedlings of the WT and the *bip3-1* mutant (**Figure 3A**) treated with TM for 5, 14, and 24 h, then performed immunoblot analysis. The *bip3-1* mutant was previously reported as a null mutant (Maruyama et al., 2010). As shown in **Figure 3B**, a specific band at approximately 75 kDa was detected at 5 h after TM treatment in the WT, whose intensity was further increased at 14 h and 24 h. Because these bands were not detected in TM-treated *bip3-1* mutant, the result indicated that anti-BiP3 antibodies we raised recognized BiP3 specifically. We detected BiP3 in roots as well as in the whole seedlings treated with TM for 10 h (**Figure 3C**). Thus, both roots and whole seedlings accumulate endogenous BiP3 in response to TM treatment.

### Spatiotemporal ER Stress Response in Arabidopsis Roots

**FIGURE 4:**
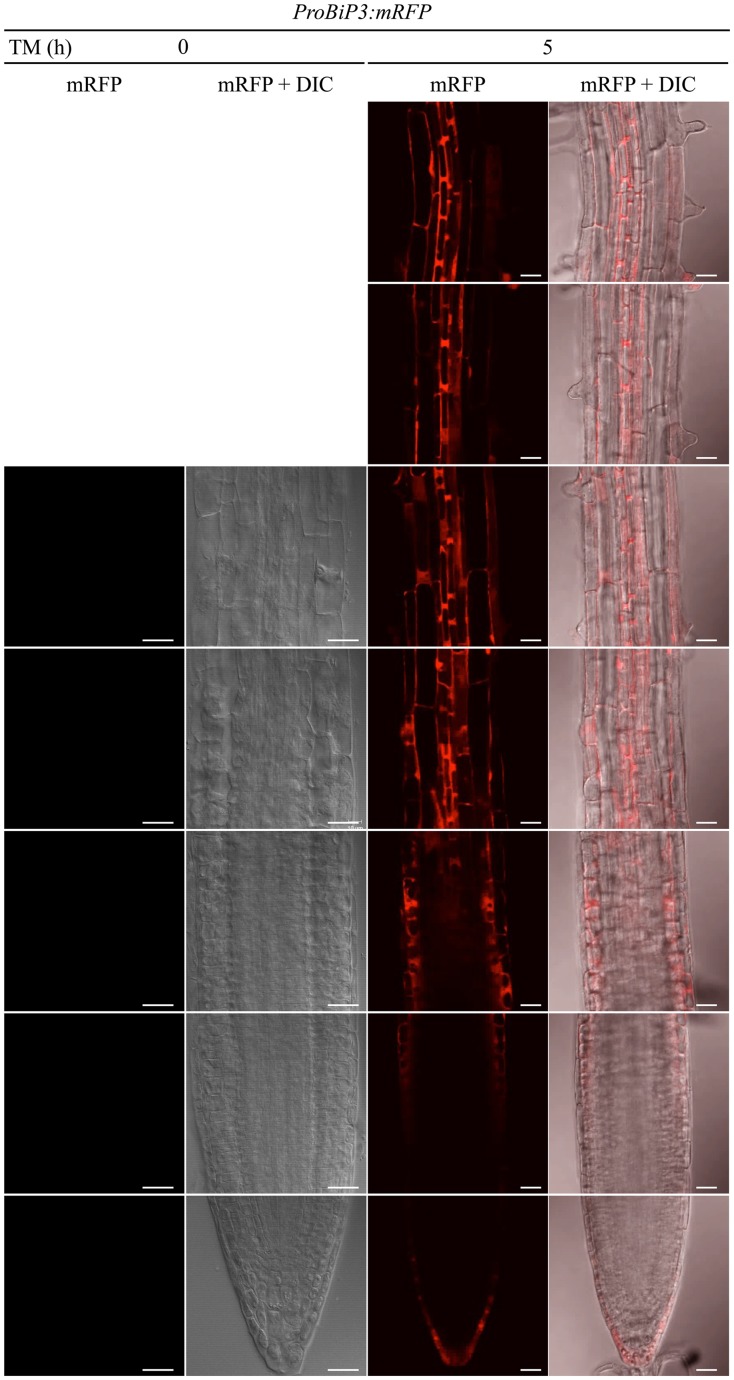
**Spatiotemporal ER stress response in Arabidopsis roots.** Observation of the *ProBiP3:mRFP* signal in roots of 7-day-old seedlings treated with 5 μg/ml TM for the indicated time. The merged images of mRFP fluorescence and DIC are shown. The fluorescent images were provided at the left side of each merged image. Scale bars, 10 μm.

Next, to observe spatiotemporal ER stress responses in intact Arabidopsis roots, we established a transgenic Arabidopsis plant that stably expresses a fluorescent mRFP protein under endogenous *BiP3* promoter (*ProBiP3:mRFP*) in the WT background. The mRFP protein was expected to localize at cytoplasm. No mRFP signals were observed in roots without the TM treatment (at 0 h in **Figure 4**). Time-course observation of the *ProBiP3:mRFP* plants up to 5 h after TM treatment showed that fluorescent signal first appeared at 3.5 h (**Figure 4** and **Supplementary Figure S2**). At 5-h treatment, obvious signals were detected into the three regions, a root tip in the meristematic zone, an outer layer in the elongation zone and an inner layer in the mature zone (**Figure 4**). This pattern became more obvious at 8-h treatment (**Supplementary Figure S3**). At 24 h after TM treatment, fluorescent mRFP signals were strongly detected at inner layers including stele in the mature zone, columella and lateral root cap of the root tip (**Supplementary Figure S3**) in the meristematic zone. Since we cannot exclude a possibility that time-dependent changes and cell type-specific features of BiP3 detection might in part reflect the kinetics of TM uptake by intact plantlets, we compared mRFP signals of the *ProBiP3:mRFP* plants treated with dithiothreitol (DTT), another known ER stress inducer. As shown in **Supplementary Figure S4**, the patterns with DTT treatment were similar to those with TM at 5- and 8-h treatments although the intensity of signal was slightly lower than that with TM. These localizations are in agreement with the result of GUS staining (**Figure 1**) at the early time point after TM treatment. Nevertheless, the other cell did not show the mRFP signal at 24 h, suggesting that specific cells may respond to the ER stress in Arabidopsis roots.

### Distribution of Misfolded Protein via Detection of Aggresome in Arabidopsis Roots

**FIGURE 5:**
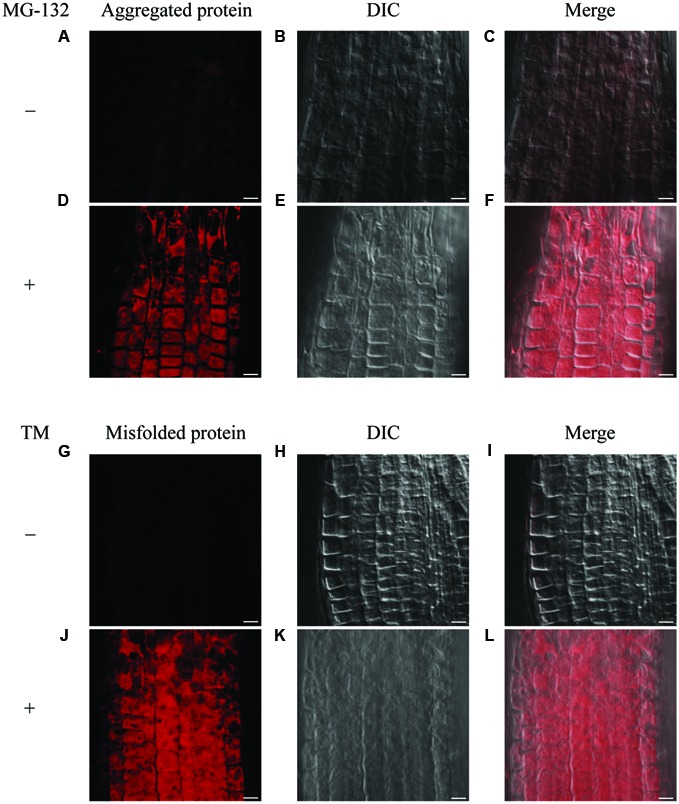
**Detection of aggregated proteins and misfolded proteins in roots. (A–F)** Detection of aggregated proteins using MG-132 in meristematic zone of roots. Seven-day-old WT seedlings were treated with or without MG-132 for 16 h. **(G–L)** Detection of misfolded proteins after TM treatment in meristematic zone of roots. Seven-day-old WT seedlings were treated with or without TM for 8 h. Staining of aggregated or misfolded proteins **(A,D,G,J)**, DIC images **(B,E,H,K)**, and merged images were shown **(C,F,I,L)**. Scale bars, 10 μm.

It has been known that the accumulation of misfolded protein in the ER triggers the ER stress response. To investigate the distribution of misfolded protein in Arabidopsis roots under ER stress conditions, we first tested detection of an aggresome by use of a commercially available aggresome detection kit (Proteostat^®^ Aggresome Detection Kit, Enzo), which was previously reported to detect aggresome formed by adding proteasome inhibitor MG-132 in mammalian cells as well as plant cultured cells (Kothawala et al., 2012; Nakajima and Suzuki, 2013). When we treated 7-day-old WT seedlings with MG-132 for 16 h, signals for aggregation were detected in root epidermis cells (**Figures 5D–F** and **Supplementary Figures S5A–C**), but not in the cells with mock treatment (**Figures 5A–C**). This result indicates that the assay works for the intact Arabidopsis roots. Next, we treated 7-day-old WT seedlings with TM to examine whether this assay detects misfolded proteins under the ER stress condition. As can be seen in (**Figures 5J–L** and **Supplementary Figures S5D–F**), the signals were detected in root epidermal cells treated with TM, but not without the treatment (**Figures 5G–I**). Hence, the assay can be used for the detection of misfolded proteins in Arabidopsis roots.

### Localization of BiP3 and Misfolded Proteins under the ER Stress Condition

**FIGURE 6:**
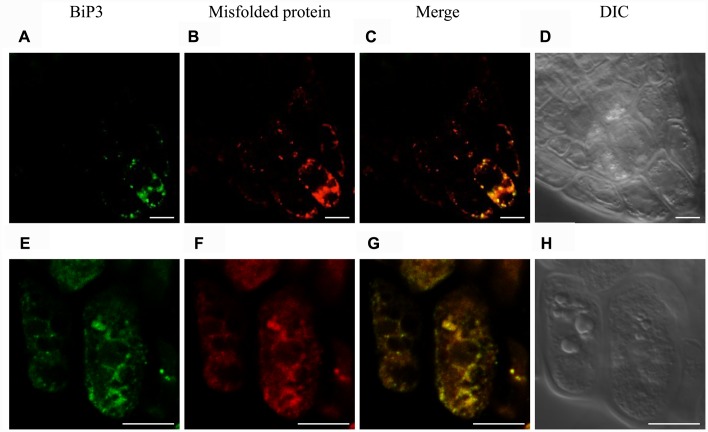
**Immunofluorescence analysis of BiP3 and colocalization with misfolded proteins in roots.** Four-day-old WT seedlings were treated with TM for 24 h. BiP3 localization detected by anti-BiP3 antibodies **(A,E)**, misfolded proteins detected by Aggresome dye ProteoStat^®^ **(B,F)**, merged images **(C,G)**, and DIC images **(D,H)**. Scale bars, 10 μm.

To observe a possible co-localization of misfolded protein and BiP3 under the ER stress in Arabidopsis roots, we performed immunofluorescence analysis using anti-BiP3 antibodies and fluorescent-labeled secondary antibodies with the above-mentioned assay to detect misfolded proteins. When the seedlings were treated with TM for 24 h, BiP3 and misfolded proteins co-localized well at columella in root tips (**Figures 6C,G** and **Supplementary Figure S6**), indicating the co-localization of ER stress response represented by BiP3 and misfolded protein accumulation.

## Discussion

Current study explored ER stress response in roots of Arabidopsis. *BiP3* was more rapidly and intensively induced in root tissues compared to leaves (**Figure 1**). Indeed, the induction of *BiP3* was more significant in roots for not only *BiP3* but also *BiP1/2* after 2 h of TM treatment (**Figure 2**). This rapid up-regulation was also found for *CNX1* (**Figure 2**). Between 2 h and 5 h after TM treatment, the relative fold changes of UPR genes expression were less significant in roots than whole seedlings. Moreover, high abundance in BiP3 proteins was detected in roots after 10 h of ER stress treatment (**Figure 3C**). Of note, no obvious morphological changes were found in roots during the short-term TM treatment (**Figure 4** and **Supplementary Figure S2**), while the long-term TM treatment is known to cause the root growth defect (Cho et al., 2015; Kanehara et al., 2015b).

Interestingly, *BiP3* was not ubiquitously expressed among different tissues in young seedlings under the ER stress condition. Our GUS reporter assay revealed that BiP3 is primarily expressed in vascular tissues and apical meristem in roots as well as hydathodes in leaves (**Figure 1**). This observation in roots was further elaborated by *BiP3* promoter reporter assay with mRFP, which not only supports the result of GUS staining but also detailed the location of *BiP3* expression at root tips in the meristematic zone, the outer layer in the elongation zone and the inner layer in the mature zone (**Figure 4**). In addition, the co-localization of misfolded proteins and BiP3 was observed in the root tip (**Figure 6**). Although hydathodes, root caps, and root apical meristems are known for their secretory activity, root hair cells that are another highly secretory cells did not show a high *BiP3* expression to the TM-induced ER stress in our observation (Battey et al., 1996; Tsugeki and Fedoroff, 1999; Komarnytsky et al., 2000; Pilot et al., 2004; Preuss et al., 2004; Cole et al., 2014). This suggests that cell-type specific responses do not simply reflect different secretory activity of individual cell types.

**FIGURE 7:**
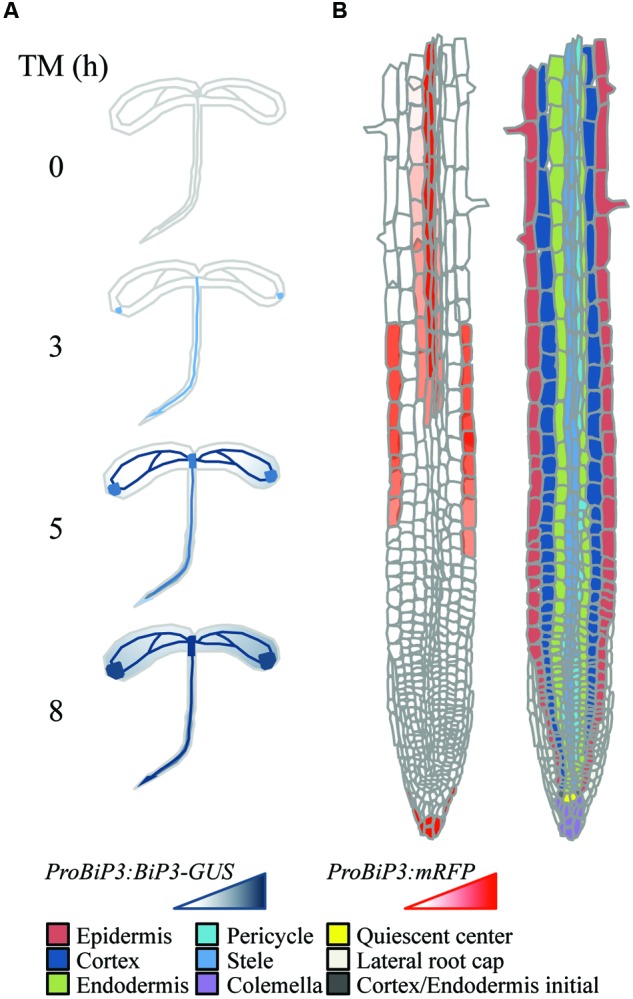
**Schematic representation of tissue-specific ER stress response in Arabidopsis roots. (A)** Expression patterns of *ProBiP3:BiP3-GUS* reporter in 7-day-old seedlings at 0, 3, 5, and 8 h after TM treatment. **(B)** Spatial expression patterns of *ProBiP3:mRFP* reporter in 7-day-old seedlings in response to TM treatment (left). Right-side diagram illustrates classification of cell types in roots.

Taken together these observations, we propose a schematic model in which a specific response to ER stress is suggested based on the reporter assay of BiP3 following TM treatment at tissue (**Figure 7A**) or cellular (**Figure 7B**) levels. Molecular mechanisms on the ER stress response have been extensively studied in unicellular models; however, how individual tissues or cells are orchestrated in intact multicellular organisms is an enigma to date. This specific response to TM may correspond to the differential strategy or priority of ER stress response among different tissues, whose mechanistic details await future investigation based on our current study. In conclusion, we suggest that ER stress response in roots has tissue specificity.

## Author Contributions

KK conceived the research and designed the experiments; YC performed the experiments and analyzed the data; YC and KK wrote the manuscripts; both authors commented on the manuscript and approved the contents.

## Conflict of Interest Statement

The authors declare that the research was conducted in the absence of any commercial or financial relationships that could be construed as a potential conflict of interest.

## Footnotes

1 http://clustalw.ddbj.nig.ac.jp/

2 http://www.ch.embnet.org/software/BOX_form.html

## Supplementary Material

FIGURE S1 **Multiple alignment of amino acid sequences of the BiPs.** The carboxyl-terminal sequences used to raise BiP3-specific antibodies are underlined.

FIGURE S2 **Spatiotemporal ER stress response in Arabidopsis roots.** Observation of the *ProBiP3:mRFP* signal in roots of 7-day-old seedlings treated with 5 μg/ml TM for the indicated time. The merged images of mRFP fluorescence and DIC are shown. Scale bars, 10 μm.

FIGURE S3 **Spatiotemporal ER stress response in Arabidopsis roots.** Observation of the *ProBiP3:mRFP* signal in roots of 7-day-old seedlings treated with 5 μg/ml TM for the time indicated. Merged images of mRFP fluorescence and DIC are shown. The fluorescent images were provided at the left side of each merged image. Scale bars, 10 μm.

FIGURE S4 **Spatiotemporal ER stress response in Arabidopsis roots treated with DTT.** Observation of the *ProBiP3:mRFP* signal in roots of 7-day-old seedlings treated with 5 μg/ml TM or 2 mM dithiothreitol (DTT) for the time indicated. Merged images of mRFP fluorescence and DIC are shown. The fluorescent images were provided at the left side of each merged image. Scale bars, 10 μm.

FIGURE S5 **Detection of aggregated proteins and misfolded proteins in roots. (A–C)** Detection of aggregated proteins using MG-132 in roots. Seven-day-old WT seedlings were treated with MG-132 for 16 h. **(D–F)** Detection of misfolded proteins after TM treatment in roots. Seven-day-old WT seedlings were treated with TM for 8 h. Staining of aggregated or misfolded proteins **(A,D)**, DIC images **(B,E)**, and merged images **(C,F)**. Scale bars, 10 μm.

FIGURE S6 **Immunofluorescence analysis of BiP3 and colocalization with misfolded proteins in roots.** Four-day-old WT seedlings were treated with TM for 24 h. BiP3 localization detected by anti-BiP3 antibodies **(A)**, misfolded proteins detected by Aggresome dye ProteoStat^®^ **(B)**, merged image **(C)**, and DIC image **(D)**. Scale bars, 10 μm.

## Abbreviations

ER: stress, endoplasmic reticulum stress
TM: tunicamycin
BiP: immunoglobulin-binding protein
UPR: unfolded protein response.
