# Supplementary figures and images for: Endoplasmic Reticulum Stress Response in Arabidopsis Roots
Source: Front Plant Sci. 2017 Mar 1;8:144. doi: 10.3389/fpls.2017.00144 (PMC5331042; doi:10.3389/fpls.2017.00144)

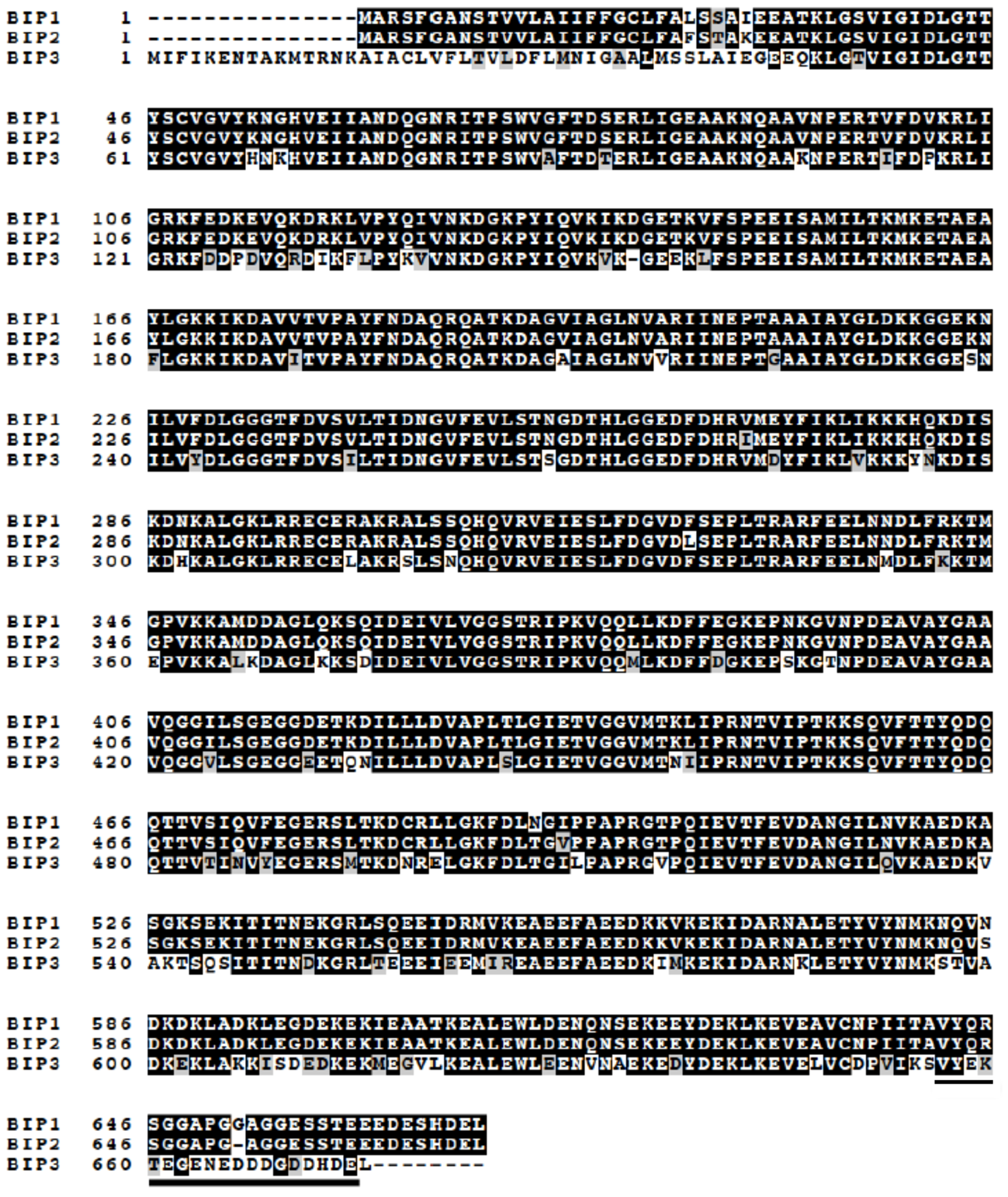

Supplement: FIGURE S1 — Multiple alignment of amino acid sequences of the BiPs. The carboxyl-terminal sequences used to raise BiP3-specific antibodies are underlined. [file Image_1.TIF]

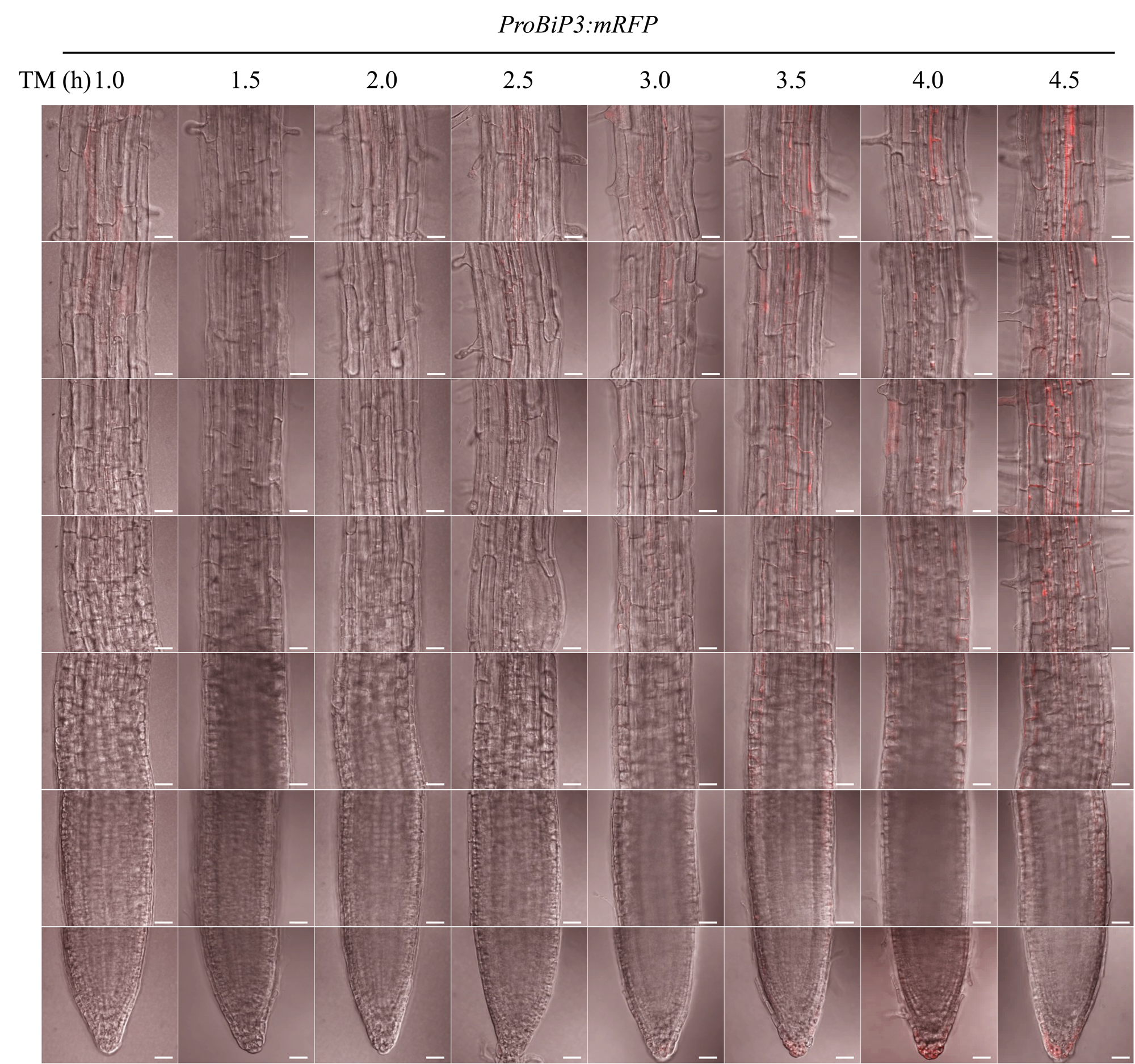

Supplement: FIGURE S2 — Spatiotemporal ER stress response in Arabidopsis roots. Observation of the ProBiP3:mRFP signal in roots of 7-day-old seedlings treated with 5 μg/ml TM for the indicated time. The merged images of mRFP fluorescence and DIC are shown. Scale bars, 10 μm. [file Image_2.TIF]

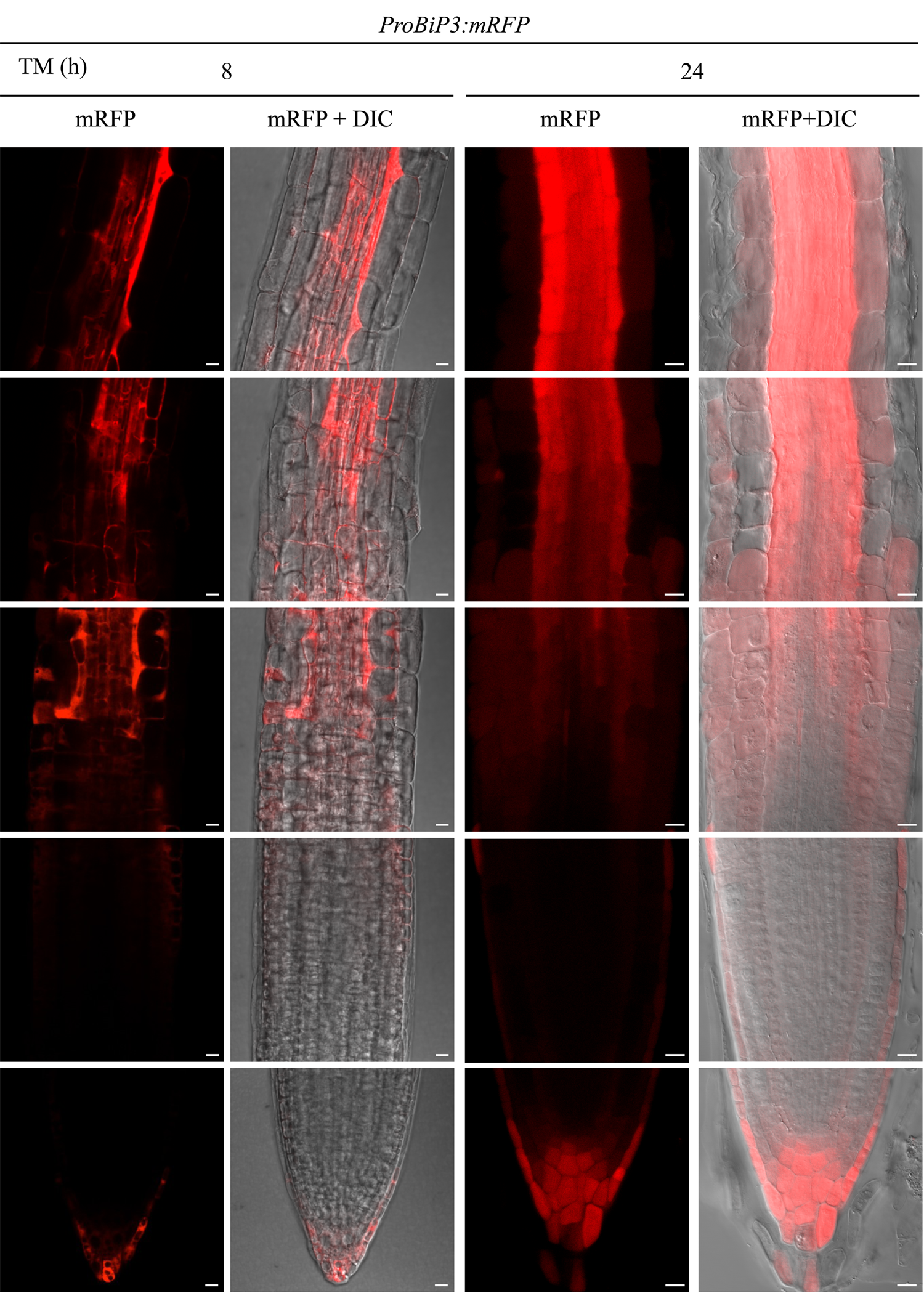

Supplement: FIGURE S3 — Spatiotemporal ER stress response in Arabidopsis roots. Observation of the ProBiP3:mRFP signal in roots of 7-day-old seedlings treated with 5 μg/ml TM for the time indicated. Merged images of mRFP fluorescence and DIC are shown. The fluorescent images were provided at the left side of each merged image. Scale bars, 10 μm. [file Image_3.TIF]

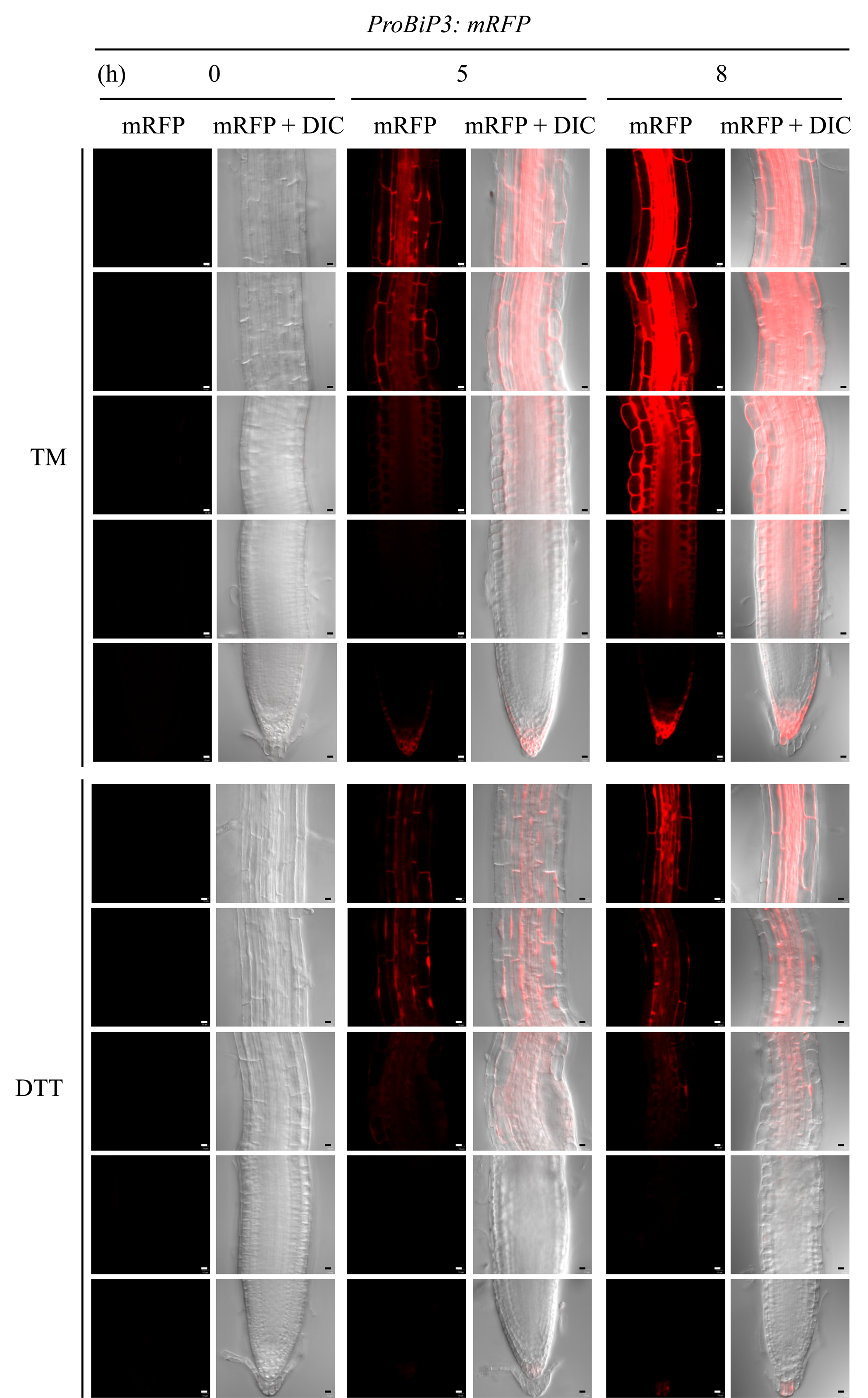

Supplement: FIGURE S4 — Spatiotemporal ER stress response in Arabidopsis roots treated with DTT. Observation of the ProBiP3:mRFP signal in roots of 7-day-old seedlings treated with 5 μg/ml TM or 2 mM dithiothreitol (DTT) for the time indicated. Merged images of mRFP fluorescence and DIC are shown. The fluorescent images were provided at the left side of each merged image. Scale bars, 10 μm. [file Image_4.TIF]

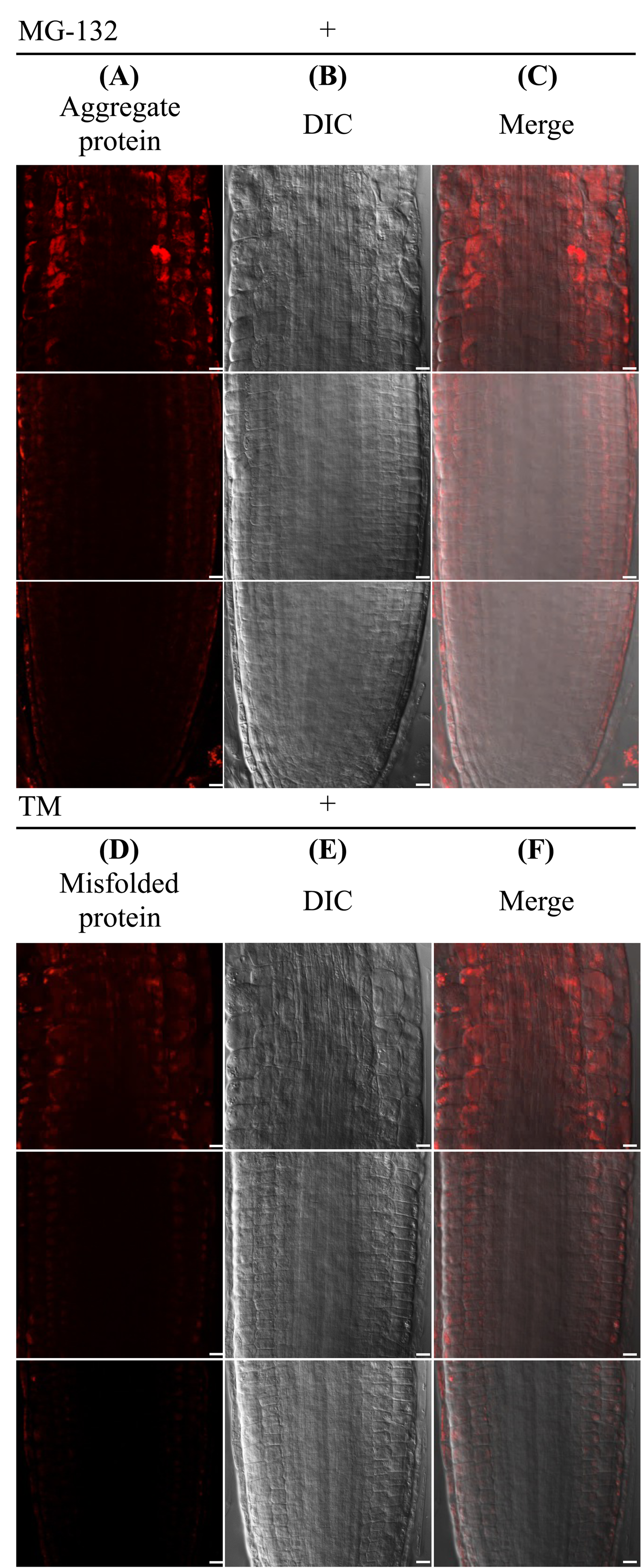

Supplement: FIGURE S5 — Detection of aggregated proteins and misfolded proteins in roots. (A–C) Detection of aggregated proteins using MG-132 in roots. Seven-day-old WT seedlings were treated with MG-132 for 16 h. (D–F) Detection of misfolded proteins after TM treatment in roots. Seven-day-old WT seedlings were treated with TM for 8 h. Staining of aggregated or misfolded proteins (A,D), DIC images (B,E), and merged images (C,F). Scale bars, 10 μm. [file Image_5.TIFF]

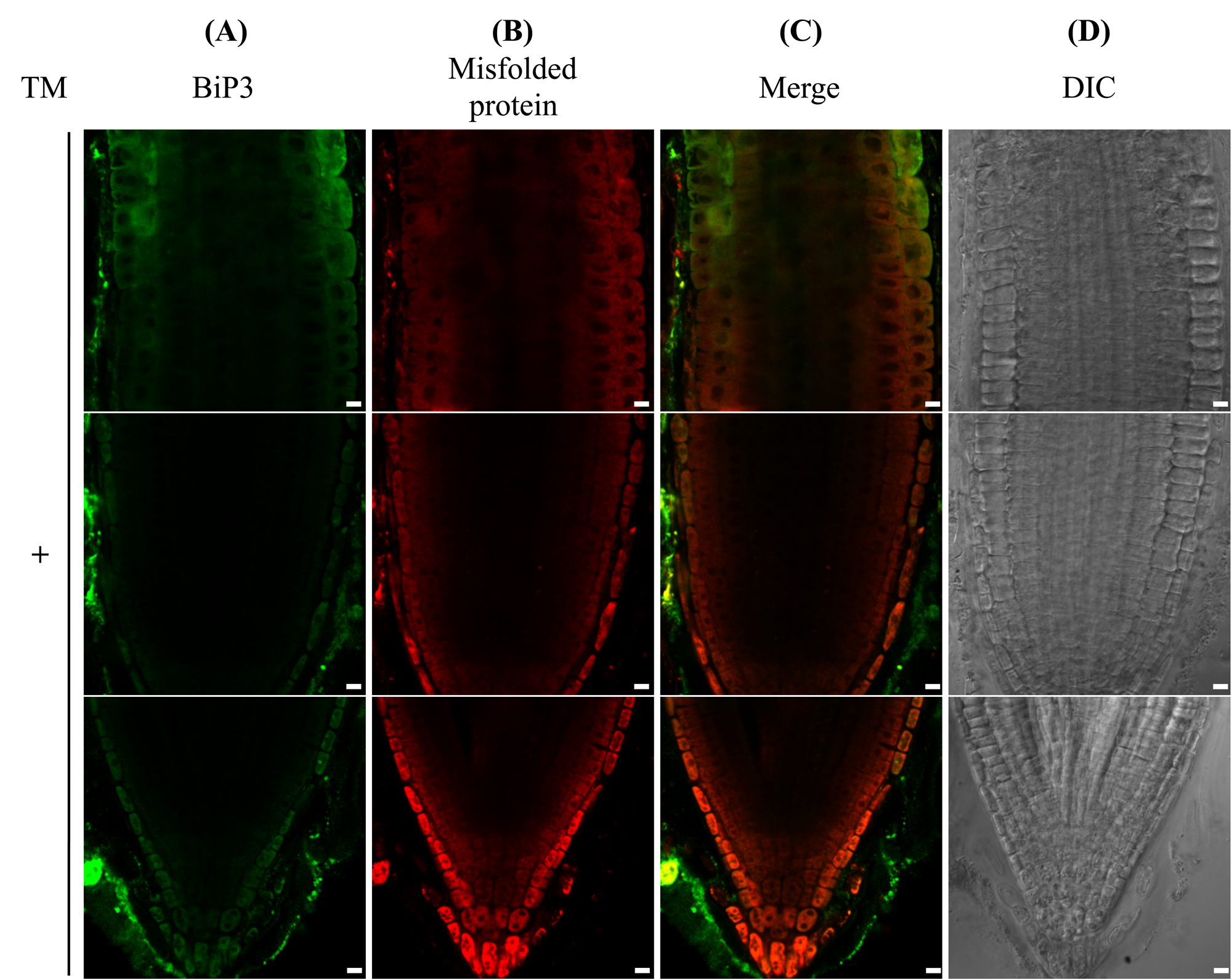

Supplement: FIGURE S6 — Immunofluorescence analysis of BiP3 and colocalization with misfolded proteins in roots. Four-day-old WT seedlings were treated with TM for 24 h. BiP3 localization detected by anti-BiP3 antibodies (A), misfolded proteins detected by Aggresome dye ProteoStat® (B), merged image (C), and DIC image (D). Scale bars, 10 μm. [file Image_6.TIFF]
