# Supplementary material for: Endoplasmic Reticulum Stress Response in Arabidopsis Roots
Source: Front Plant Sci. 2017 Mar 1;8:144. doi: 10.3389/fpls.2017.00144 (PMC5331042; doi:10.3389/fpls.2017.00144)
Supplement: Supplementary file 7 [file Table_1.PDF]

The Supplemental Table 1. List of oligonucleotide primers used in this study

Name	Gene	Purpose	Sequence (5' to 3')
KK200	BiP3	genotyping	TGGTGAAGGTGGAGAAGAAAC
KK201	BiP3	genotyping	GCCATGCCATAGAAAAGTTCC
LBb1.3	BiP3	genotyping	ATTTTGCCGATTTCGGAAC
KK131	genomic BiP3/Promoter of BiP3	Cloning	cacctcactacaattacattattcacgctg
KK132	genomic BiP3	Cloning	GTGACATGGGTGGTTTCGATTTCTGAttgc
KK152	Insertion of SmaI site in BiP3	Quick change	GAATGAGGACGACGATGGAGATGATcccgggCACGATGAGTTATAGaatcgtataattg
KK98	ProBiP3-BiP3-GUS-HDEL in pBGW	genotyping	CACTTCCTGATTATTGACCCACACTTTGCCG
KK172	Promoter of BiP3	Cloning	GGCGCCCATttttcgttgttgagaactcttcttcg
KK202	ProBiP3-mRFP in pGWB653	genotyping	cttcgctattacgccagctggcgaaag
KK133	ProBiP3-mRFP in pGWB653	genotyping	ggtgtaatatttggaagaacatgactaatgc
KK114	BiP3	qRT-PCR	CGAAACGTCTGATTGGAAGAA
KK115	BiP3	qRT-PCR	GGCTTCCCATCTTTGTTCAC
KK112	BiP1/2	qRT-PCR	TCAGTCCTGAGGAGATTAGTGCT
KK113	BiP1/2	qRT-PCR	TGCCTTTGAGCATCATTGAA
KK125	CRT1	qRT-PCR	AGACCTTAGTCTTCCAATTCTC
KK126	CRT1	qRT-PCR	CCATTGTAAGTAAGGATAGCATG
KK127	CNX1	qRT-PCR	ATGAGACAACGGCAACTATTTTCC
KK128	CNX1	qRT-PCR	CCATAATCCTCATGTCCTTCACT
KK129	ACTIN	qRT-PCR	ggtaacattgtgctcagtggtgg
KK130	ACTIN	qRT-PCR	aacgaccttaatcttcatgctgc
